# High-throughput mRNA and miRNA profiling of epithelial-mesenchymal transition in MDCK cells

**DOI:** 10.1186/s12864-015-2036-9

**Published:** 2015-11-16

**Authors:** Priyank Shukla, Claus Vogl, Barbara Wallner, Doris Rigler, Mathias Müller, Sabine Macho-Maschler

**Affiliations:** Institute of Animal Breeding and Genetics, University of Veterinary Medicine Vienna, Vienna, Austria

**Keywords:** MDCK, Epithelial-mesenchymal transition, Ras, Next generation sequencing, Transcriptome, miRNAome

## Abstract

**Background:**

Epithelial-mesenchymal transition (EMT) is an important process in embryonic development, especially during gastrulation and organ formation. Furthermore EMT is widely observed in pathological conditions, e.g., fibrosis, tumor progression and metastasis. Madin-Darby Canine Kidney (MDCK) cells are widely used for studies of EMT and epithelial plasticity. MDCK cells show an epithelial phenotype, while oncogenic Ras-transformed MDCK (MDCK-Ras) cells undergo EMT and show a mesenchymal phenotype.

**Methods:**

RNA-Seq and miRNA-Seq analyses were performed on MDCK and MDCK-Ras cells. Data were validated by qRT-PCR. Gene signature analyses were carried out to identify pathways and gene ontology terms. For selected miRNAs target prediction was performed.

**Results:**

With RNA-Seq, mRNAs of approximately half of the genes known for dog were detected. These were screened for differential regulation during Ras-induced EMT. We went further and performed gene signature analyses and found Gene Ontology (GO) terms and pathways important for epithelial polarity and implicated in EMT. Among the identified pathways, TGFβ1 emerged as a central signaling factor in many EMT related pathways and biological processes. With miRNA-Seq, approximately half of the known canine miRNAs were found expressed in MDCK and MDCK-Ras cells. Furthermore, among differentially expressed miRNAs, miRNAs that are known to be important regulators of EMT were detected and new candidates were predicted. New dog miRNAs were discovered after aligning our reads to that of other species in miRBase. Importantly, we could identify 25 completely novel miRNAs with a stable hairpin structure. Two of these novel miRNAs were differentially expressed. We validated the two novel miRNAs with the highest read counts by RT-qPCR. Target prediction of a particular novel miRNA highly expressed in mesenchymal MDCK-Ras cells revealed that it targets components of epithelial cell junctional complexes. Combining target prediction for the most upregulated miRNAs and validation of the targets in MDCK-Ras cells with pathway analysis allowed us to identify two novel pathways, e.g., JAK/STAT signaling and pancreatic cancer pathways. These pathways could not be detected solely by gene set enrichment analyses of RNA-Seq data.

**Conclusion:**

With deep sequencing data of mRNAs and miRNAs of MDCK cells and of Ras-induced EMT in MDCK cells, differentially regulated mRNAs and miRNAs are identified. Many of the identified genes are within pathways known to be involved in EMT. Novel differentially upregulated genes in MDCK cells are interferon stimulated genes and genes involved in Slit and Netrin signaling. New pathways not yet linked to these processes were identified. A central pathway in Ras induced EMT is TGFβ signaling, which leads to differential regulation of many target genes, including miRNAs. With miRNA-Seq we identified miRNAs involved in either epithelial cell biology or EMT. Finally, we describe completely novel miRNAs and their target genes.

**Electronic supplementary material:**

The online version of this article (doi:10.1186/s12864-015-2036-9) contains supplementary material, which is available to authorized users.

## Background

During development, epithelial-mesenchymal transition (EMT) and the reverse process of mesenchymal-epithelial transition (MET) are important for the spatial and temporal distribution of cells within the embryo and for proper organ formation [1]. After terminal differentiation, mesenchymal or epithelial tissue states are generally stable. Epithelial cells are immotile, show a clear apico-basal polarity, contact the basal membrane, and maintain tight cell-cell contacts laterally. On the other hand, mesenchymal cells do not show apico-basal polarity, favour cell-matrix interactions instead of cell-cell or basement membrane contacts, and are often motile and invasive. Under special circumstances epithelial cells acquire mesenchymal characteristics; this transdifferentiation process is referred to as EMT. In adult tissues, EMT occurs physiologically in, e.g., wound healing and pathologically in, e.g., organ fibrosis and cancer. In late stage tumorigenesis, cells that underwent EMT are motile and may invade other parts of the body to form distant metastases [2].

On the molecular level, EMT is defined by the loss of expression of epithelial and polarity genes, e.g., E-Cadherin and tight junction proteins, and the *de novo* expression of mesenchymal marker genes, e.g., Vimentin, Fibronectin and N-Cadherin [3]. E-Cadherin is a marker gene of epithelial cells and an important component of the adherens junction complex [4]. Expression of E-Cadherin is repressed by EMT-specific transcription factors (EMT-TF) [5]. Many signaling pathways inducing EMT converge on the transcriptional level to downregulate E-Cadherin expression and can act either synergistically or on their own to induce EMT. TGFβ/Smad signaling is prominent in EMT [6]. Furthermore, activation of receptor tyrosine kinase (RTK) signaling by either the ligand or by a mutation activating the receptor constitutively leads to EMT [7]. RTKs act upstream of Ras signaling and thereby influence cellular behavior including migration, growth and differentiation. Furthermore, oncogenic Ras signaling induces EMT in different cell types in the presence of TGFβ1 signaling [8–11]. Among other functions, TGFβ1 stimulates the synthesis of many extracellular matrix (ECM) proteins and matrix remodeling enzymes.

ECM proteins are not just static substrates for cells; rather, ECM components signal by binding to integrins located in the cell membrane [12]. Integrins are heterodimers composed of alpha and beta subunits, which activate downstream signaling upon ligand binding. This signaling regulates, e.g., cell differentiation, proliferation, apoptosis, cell adhesion, migration and invasion [13]. Changes in the expression and surface localisation of integrins during EMT have been documented [14, 15].

Another pathway capable of inducing EMT is the WNT/β-Catenin pathway [7]. WNTs are secreted growth factors binding to cell surface receptors of the frizzled family. Activated WNT signaling then stabilizes β-Catenin which translocates to the nucleus and stimulates gene expression via LEF/TCF transcription factors [16].

Besides these signaling pathways, other processes regulating gene expression are important in EMT. miRNAs influence protein expression and thereby the state of a cell. They are important for maintaining the normal physiological properties of cells [17]. Furthermore, involvement of miRNAs has also been studied in pathological situations, e.g., in fibrosis or cancer [18–20]. As noted above, the impact of EMT on these pathologies is well accepted and miRNAs regulating EMT have been identified [21, 22].

Generally, fibrosis is a disease of a tissue involving stromal and immune cells, which are activated and secrete factors (including TGFβ1) that induce cells to massively deposit ECM components. It is well known that EMT plays a crucial role in fibrosis [23–25], since a part of the fibroblastoid cells found in fibrotic tissues arise from epithelial cells that underwent EMT [26].

Recently, miRNAs driving fibrosis have been identified [18, 27, 28]. These so called fibromiRs include pro-fibrotic and anti-fibrotic miRNAs. In the context of EMT, changes in the expression of some of these miRNAs have also been described and reviewed [29, 30]. Especially members of the miR-200 family (miR-200a/b and miR-141) act via blocking pro-fibrotic and pro-EMT TGFβ1 signaling. Negative feedback loops of miRNAs and EMT-TFs have been shown for members of the miR-200 family and ZEB1 and ZEB2 [31], and for miR-203 and SNAI1 [32]. Recently, the effect of exogenous expression of EMT-TFs in MDCK cells on the expression of miRNAs has been shown [33].

Since *in vivo* EMT is a complex process, *in vitro* cell systems have been employed to study EMT. A system to study epithelial polarity and plasticity is the Madin-Darby Canine Kidney (MDCK) cell line [34, 35].

MDCK cells, isolated from the distal tubule of the kidney nephron, have been used as a model to study EMT. Several ways of inducing EMT in MDCK cells have been documented [36–39]. Specifically, Ha-Ras transformed MDCK (MDCK-Ras) cells undergo EMT in the presence of TGFβ signaling [15, 40, 41]. This has been used to study different aspects of this process including plasma membrane remodelling [15], extracellular matrix composition, changes in the lipid composition of the plasma membrane [42] and secreted factors [40, 41]. Changes in the composition of exosomes have also been reported [43].

Previously, microarray technology has been used to obtain mRNA and miRNA expression patterns of MDCK cells and growth factor induced phenotypic changes of MDCK cells [44–46, 33]. Since microarrays cover only the subset of probes present on the array they thus provide an incomplete picture of the changes in gene expression during EMT. On the other hand, large scale next generation sequencing provides unbiased data to identify novel genes and gene sets important to EMT.

In this article, we used the Next Generation Sequencing (NGS) technology to complement and extend the transcriptome and miRNAome of epithelial MDCK cells and mesenchymal MDCK-Ras cells. In addition, we provide gene signature analysis using GO categories and pathways enriched with differentially expressed genes. Further we present completely novel miRNAs and provide information on miRNAs not yet linked to EMT and discuss their targets. Thus we increase knowledge on the transcriptional landscape of mRNAs and miRNAs in MDCK cells and in MDCK cells that underwent EMT induced by oncogenic Ras.

## Methods

### Cell culture

Since several divergent strains of MDCK cells exist, it is important to specify the particular strain used for analysis [47]. In our study we used MDCK type II.

The canine origin of MDCK and MDCK-Ras cells (MDCK cells expressing oncogenic Ha-Ras) was confirmed by a species-specific PCR restriction fragment length polymorphism pattern (RFLP; Additional file 1: Figure S1A). Expression of V12-Ha-Ras in MDCK-Ras cells was confirmed by immunoprecipitation (IP) of Ha-Ras followed by immunoblot for pan-RasV12 (Additional file 1: Figure S1B). MDCK and MDCK-Ras cells (both from H. Beug, IMP Vienna; [48]) were cultivated in DMEM/F12 (Gibco) high glucose medium supplemented with 10 % FCS (PAA), 2 mM L-Glutamine (PAA), 10 mM HEPES pH7, 100 I.U Penicillin (PAA) and 100 μg/ml Streptomycin (PAA) in a humified incubator at 5 % CO_2_. Cells were grown to confluency, washed with 1xPBS (Sigma), scraped off, resuspended in 1xPBS and centrifuged at 1500 rpm for 5 min. The resulting cell pellet was stored at −80 °C and used for further analysis. All samples of MDCK and MDCK-Ras cells tested negative for mycoplasma infection by PCR using the Venor®GeM Classic PCR-Kit (Minerva Biolabs; #11-1050) according to the manufacturer’s instructions. Reporter gene assays were performed as described in [49].

### Restriction fragment length polymorphism analysis

Following DNA isolation, a single fragment in the mitochondrial 16S rRNA gene was amplified using the primers 16S uni F (5’- TAA CGA GCC TGG TGA TAG CTG) and 16S uni R (5’- GAT TAT GCT ACC TTT GCA CGG T). PCR was performed in a final volume of 25 μL, containing 200 μM each dNTP, 1.5 mM MgCl_2_, 500nM each primer, 1U Taq polymerase and DNA according to availability in 1xPCR buffer. Amplification was carried out after an initial denaturation at 95 °C for 5 min for 35 cycles (94 °C for 30 s, 52 °C for 40 s, 72 °C for 40 s), followed by a final extension at 72 °C for 5 min. A multiple restriction digest (*Vsp*I, *Hind*III and *Hinf*I) of 20 μL PCR product was performed in a final volume of 30 μL 1x restriction buffer, containing 1U of each restriction enzyme. The reaction was incubated over night at 37 °C and subsequently loaded onto a 2 % agarose gel.

### Protein analysis

For protein extraction, cells were washed with ice-cold PBS and lysed for 20 min on ice with RIPA (RadioImmunoPrecipitationAssay) buffer (150 mM NaCl, 50 mM Tris pH7.4, 1 % NP40, 1 % sodium-deoxycholat, 1 mM EDTA, 1 mM Na_3_VO_4_, 25 mM NaF, 1 mM PMSF, 5 mM beta-glycerolphosphat, protease inhibitor cocktail tablets (Complete mini; Roche)). Total cell lysates were cleared by centriguation (10 min, 4 °C, 10,000 g). Protein concentration was measured with the Bradford ProteinAssay (BioRad). Western blots were performed as described in [50] with the following antibodies: anti-Fibronectin: Santa Cruz, sc-9068; anti-ZO1: Zymed Laboratories, 33–9100; anti-E-Cadherin: BD Transduction Laboratories, 610181; anti-Vimentin: Sigma, V2258; anti-Actin: Sigma, A2066; anti-phospho-AKT (Ser473): Cell Signaling, 9271; anti-total-AKT: Cell Signaling, 9272; anti-phospho-ERK1/2: Sigma, M8159; anti-total-ERK1/2:Sigma,M5670; anti-Pan-Ras: Calbiochem, OP38.

For immunoprecipitation, cells were lysed as described for Western blotting. Equal amounts of protein were incubated with anti-v-H-Ras antibody (Calbiochem, OP01) overnight at 4 °C. Then ProteinA/G plus beads (Santa Cruz) were added and samples were incubated for 1 h at 4 °C. Thereafter, immune complexes were collected by centrifugation, washed twice with ice cold RIPA buffer and subjected to SDS PAGE and Western blotting.

### RNA isolation, reverse transcription (RT) and quantitative real-time PCR (qPCR)

Total RNA was isolated from 4 biological replicates from each cell type using peqGOLD TriFast (Peqlab) according to the manufacturer’s instructions. Amount and quality of RNA samples were checked by spectrophotometric analysis and agarose gel electrophoresis. After a DNA digestion step (RQ1 RNAse-Free DNAse, Promega), RNA (1 μg /20 μl reaction volume) was reverse transcribed using the iScript cDNA synthesis kit (Bio-Rad, Vienna, Austria).

To evaluate epithelial and mesenchymal transcriptional characteristics of MDCK and MDCK-Ras cells, RT-qPCR of two epithelial and five mesenchymal markers was performed. 2 μl of 1:4 diluted cDNA was used in a 25 μl mastermix, containing 2.5 mM MgCl_2_, 200 nM of each dNTP (MBI Fermentas), 1 × QuantiTect primer assay (Qiagen), 0.2 × EvaGreen (Biotium), 1 Unit HotFire DNA polymerase (Solis Biodyne), and 1 × reaction buffer B (Solis Biodyne). The following cycling conditions were used on a Stratagene Mx3000P machine using: Initial denaturation at 95 °C for 15 min, 40 cycles of 95 °C for 15 s, 55 °C for 30 s and 72 °C for 30 s. Melting curve analyses were performed in order to check amplicon specificity. Assay specifications are listed in Additional file 2: S1. The data were analyzed using the Mx3000P Analysis software. The expression levels of target genes were normalised to the expression level of the reference gene GAPDH. Comparable efficiencies between target and reference genes were confirmed by calibration curves, which were also used to determine the dynamic range of the assays. Samples with Ct-values >35 were considered “not detectable”. The fold changes were obtained using the ∆∆C_t_ method [51].

Statistical analyses were performed using linear models after log transformation using the “R” programming language [52]. Genes, where all samples were above the detection limit (i.e., with a Ct < 35), were tested for significant differences between MDCK and MDCK-Ras with t-tests. For genes, where at least one sample in one of the conditions (MDCK or MDCK-Ras) was below the detection limit, the mean and its 95 % confidence interval in the condition with complete data were calculated. Based on these values a conservative test was derived: if the confidence interval did not overlap the detection limit (i.e., a delta Ct of 35 minus the maximal Ct value for the housekeeping gene), the two conditions were considered significantly different.

### RNA-Seq - library preparation and sequencing

Concentration and quality of the RNA samples from 4 biological replicates (RNA prepared as described above) per cell line (MDCK and MDCK-Ras) were determined using the Agilent Bioanalyzer according to the manufacturer’s instructions. RNA poly(A) + selected cDNA libraries were prepared from a starting amount of 15 μg total RNA following a protocol preserving the strand information based on the dUTP method [53]. Strand specific and indexed sequencing libraries with 200 – 700 bp insert size were generated using the NEBNext® Ultra™ DNA Library Prep Kit for Illumina® New England Biolabs). Each library was loaded into 4 different lanes (technical replicates) of an Illumina HiSeq 2000 flowcell. 100 bp paired-end sequencing was performed according to the manufacturer’s protocol (Illumina).

### RNA-Seq reads quality control, alignment, expression profiling and analysis of differential expression

Read quality was checked with FASTQC (http://www.bioinformatics.babraham.ac.uk/projects/fastqc). The first 10 bp at the start of each read were trimmed-off using an in-house python script, because their nucleotide (A, T, G, C) content ratio did not conform to Chargaff’s rule. At the 3’ end of each read, low quality reads were trimmed using the “trim-fastq.pl” script of the PoPoolation Toolbox [54], which is based on a modified Mott algorithm. Reads of length less than 40 bp were discarded. Quality controlled reads were mapped to the dog genome (Ensemble’s CanFam3.1; release 68) using GSNAP [55] (with the parameters: “--nthreads = 4, −d CanFam3, −D path to genome index, −-novelsplicing = 1, −-use-splicing = CanFam3_1_68_gtf_splicesitesfile and --format = sam”); the resulting alignments were saved in the SAM format. Reads from technical replicates were merged together. With SAMtools [56] uniquely mapped reads mapped in proper pairs were extracted and fed to HTSeq [57] to count reads mapped to each gene using the gene annotation file (gtf) from Ensemble (CanFam3.1; release 68). Genes with a mean mapped read count of less than 50 were considered as low expressed and discarded. Differential expression analysis was performed using DESeq [58]. The mapped read count per gene of each sample library was normalized by its respective effective library size. The variance of counts was computed as in [58]. Differential expression between the two conditions (MDCK vs. MDCK-Ras) for each gene was tested with the negative binomial test at a significance level of 0.05.

### RT-qPCR validation of RNA-Seq results

26 genes were chosen for RNA-Seq validation by RT-qPCR. Selected genes and Assays (Qiagen) are listed in Additional file 2: S1. cDNA preperation and RT-qPCR was performed on four new independent MDCK and MDCK-Ras samples as described above.

### Gene ontology (GO) analysis

GO annotations for the dog species were downloaded from the Gene Ontology consortium website (http://geneontology.org/). The average differential expression of genes (z-scores of log2 fold change) belonging to a specific GO category was tested for deviation from the average of all other genes using a z-test. A FDR ≤ 5 % was used as cutoff to select significantly enriched GO terms.

### Gene set enrichment analysis for canonical pathways

Canonical pathway gene sets of REACTOME, KEGG and PID were downloaded from MSigDB [59] and were tested using Gene Set Enrichment Analysis (GSEA) tool [59] for enrichment in our list of differentially expressed genes. Ranking of these genes was based on log2 fold change derived from DESeq. A FDR ≤ 10 % was used as cutoff to select significantly enriched pathways. For selected miRNA target genes, pathway analysis was performed via DAVID [60]. A FDR of ≤ 10 % was used as cutoff to select significantly enriched pathways.

### miRNA Sequencing- library preparation and Illumina sequencing

Quality and quantity of total RNA from MDCK and MDCK-Ras cells isolated with peqGOLD TriFast (Peqlab) were checked on an Agilent Bioanalyzer using the Agilent RNA 6000 Nano Kit (Agilent). The miRNA sequencing libraries were prepared from four biological replicates per experimental condition with the Illumina Small RNA Sample Prep v1.5 (Illumina, San Diago, CA USA) as described in the corresponding protocol. Amplified cDNA libraries were size fractionated on a 2 % low-melt agarose gel and fragments with a length between 90 to 110 nucleotides were excised. Eluated template libraries were quantified and quality checked using the Qubit dsDNA HS Assay kit (Invitrogen) and on an Agilent Bioanalyzer (Agilent). Each library was loaded into a single lane of Illumina Genome Analyzer II flowcell. 36 bp single-end sequencing was performed according to the manufacturer’s protocols (Illumina).

### miRNA-Seq reads quality control, alignment, miRNA detection and prediction, expression profiling and analysis of differential expression

Quality of reads was checked with FASTQC (http://www.bioinformatics.babraham.ac.uk/projects/fastqc), during which an overrepresentation of “Illumina Small RNA 3p Adapter 1” was found. Cutadapt [61] was used to trim this adaptor sequence from reads. At the 3’ end of each read, low quality reads were trimmed using “trim-fastq.pl” script of the PoPoolation Toolbox [54]. FASTQ files were converted into FASTA format using the FASTX-Toolkit (http://hannonlab.cshl.edu/fastx_toolkit/). Reads with a length less than 17 bp were discarded and identical reads were collapsed using the miRDeep2 [62] tool. Furthermore, reads that aligned to other non-coding RNAs (e.g., tRNA, snRNA, snoRNA, scRNA, rRNA etc.) sequences present in Rfam database (release 11.0) [63] were discarded using Bowtie [64], allowing one mismatch in the whole alignment region. Finally, these quality controlled reads were aligned to the dog genome (Ensemble’s CanFam3.1; release 68) using Bowtie, again allowing one mismatch in the whole alignment region. Using the miRDeep2 tool and miRBase (release 21), sequences were matched to miRNAs known in dog, then to miRNAs known in humans, and, subsequently, in other species. For canis, a miRDeep2 log-odds score cutoff of greater or equal to four was used, which yielded signal-to-noise ratio of at least 10:1. For all other species, the prediction of the hairpin secondary structure was also used for identification of miRNAs using the program RNAfold implemented within miRDeep2 toolkit with default options. For human specific miRNAs, also a log-odds score cutoff of four was used; for miRNAs of other species and also for novel miRNAs, a miRDeep2 log-odds score cutoff greater or equal to six was used, which yielded signal-to-noise ratio of at least 14:1. Differential expression analysis was performed as described above for RNA-Seq.

### Validation of miRNA-Seq results

For validation, miRNAs were isolated from four new independent MDCK and MDCK-Ras samples using the miRNeasy Mini Kit (Qiagen). Mature miRNAs were reverse-transcribed with the miScriptII RT-Kit using High spec buffer (1,8 μg total RNA starting material). The resulting cDNAs were diluted 1:20 and RT-qPCR was performed using miScript SYBR-Green PCR-Kit, miScript universal Primer and miScript Primer Assays (Additional file 3: S2) according to the manufacturer’s protocol on a Stratagene MX3000P (Agilent Technologies). Thereafter, miRNA expression levels were normalized to the endogenous control RNU6B. Fold changes were calculated and statistical tests performed as described in the RT-qPCR section above. Validation of the novel miRNA#1-3 was performed as described above with customised primer assays (Qiagen) designed to amplify the mature sequence of these miRNAs.

### miRNA clustering based on family and genome coordinates

Differentially expressed miRNAs were grouped according to family information and their genome coordinates (inter-miRNA distance <10 kb) using miRBase (release 21).

### miRNA target prediction

For all differentially expressed genes identified by RNA-Seq, 3’UTR sequences were downloaded from Ensemble (Canfam3.1) and the genes were tested for being targeted by the differentially expressed miRNAs using TargetScan (v6.2) perl scripts [65]. Context Specific Score (CSS) of less than −0.1 was used as a cutoff for significant target-miRNA pairs. To refine predictions, the inverse correlation between expression of a miRNA and its target mRNA was used [66].

### Data accessibility

RNA-Seq and miRNA-Seq data are available in the ArrayExpress database (www.ebi.ac.uk/arrayexpress) under accession number E-MTAB-3301 (RNA-Seq) and E-MTAB-3302 (miRNA-Seq).

## Results

### Characterisation of MDCK and MDCK-Ras cells

**Fig. 1:**
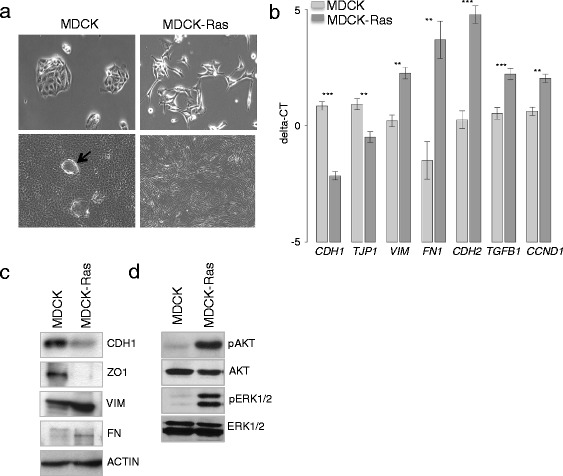
Characterization of MDCK and MDCK-Ras cells. **a** Phase contrast pictures of subconfluent (upper panel) and confluent (lower panel) cultures of MDCK (black arrow in MDCK indicate domes) and MDCK-Ras cells. **b** RT-qPCR and (**c**) Western Blot analysis of epithelial and mesenchymal markers. Actin was used as loading control. **d** Western blot analysis of pAKT, AKT, pERK1/2 and ERK1/2. Stars in (**b**) indicate significance values: ***p < 0.001; **p < 0.01

First we analysed the phenotypes of MDCK and MDCK-Ras cells (Fig. 1a). MDCK cells show an epithelial morphology and grow as epithelial island in subconfluent cultures (Fig. 1a; left upper panel). In confluent cultures they form hemicysts, typical for epithelial cells (black arrow in Fig. 1a; left lower panel). In contrast, MDCK-Ras cells display a fibroblastoid phenotype, clearly visible in subconfluent cultures (Fig. 1a; right upper panel). Confluent MDCK-Ras cells show an overgrowth phenotype without contact inhibition (Fig. 1a; right lower panel). The phenotype of MDCK-Ras cells is very similar to the phenotype described in [48].

Next we analysed mRNA and protein expression of epithelial and mesenchymal markers in six biological replicates of each cell type by RT-qPCR and Western blotting, respectively. The epithelial-specific markers E-Cadherin (*CDH1*) and Tight Junction Protein1 (*TJP1* also known as ZO1), both important components for maintaining cell-cell contacts in epithelial cells, were clearly expressed in MDCK cells and strongly reduced in mesenchymal MDCK-Ras cells (Fig. 1b, c). In contrast, expression levels of the mesenchymal markers Vimentin (*VIM*), Fibronectin (*FN1*), N-Cadherin (*CDH2*) and Transforming growth factor β1 (*TGFB1*) as well as Cyclin D1 (*CCND1*) were clearly enhanced in MDCK-Ras cells (Fig. 1b, c). Additionally we analysed Ras downstream signaling in MDCK and MDCK-Ras cells. MDCK-Ras cells display strong activation of PI3K and ERK1/2 signaling, two major downstream pathways of Ras [67]. Phosphorylation levels of AKT at Ser473 and of ERK1 and ERK2 were higher in MDCK-Ras cells compared to MDCK cells, implicating active Ras signaling (Fig. 1d).

In summary, these results confirmed the epithelial phenotype and characteristic gene expression pattern of MDCK cells, and the mesenchymal phenotype and gene expression pattern of MDCK-Ras cells. Additionally, the presence of hyper-activated Ras downstream signaling in MDCK-Ras cells was shown. We therefore proceeded with mRNA and miRNA deep sequencing.

### mRNA expression patterns in MDCK and MDCK-Ras cells

For RNA-Seq we sequenced four biological replicates of MDCK and MDCK-Ras cells each by Illumina HiSeq 2000. To avoid possible technical bias due to different lanes on the flow cell, we pooled all eight samples and sequenced them on four lanes of the same flow cell. Specific indexing adaptors were used to identify and demultiplex the eight samples later. This setup resulted in 4 technical replicates of each biological replicate; i.e., in total 32 samples. The sequencing run yielded 25–81 million 100 bp paired-end reads per biological replicate, with a total of 424 million paired-end reads (Additional file 4: Table S1).

### Read quality control and mapping

**Fig. 2:**
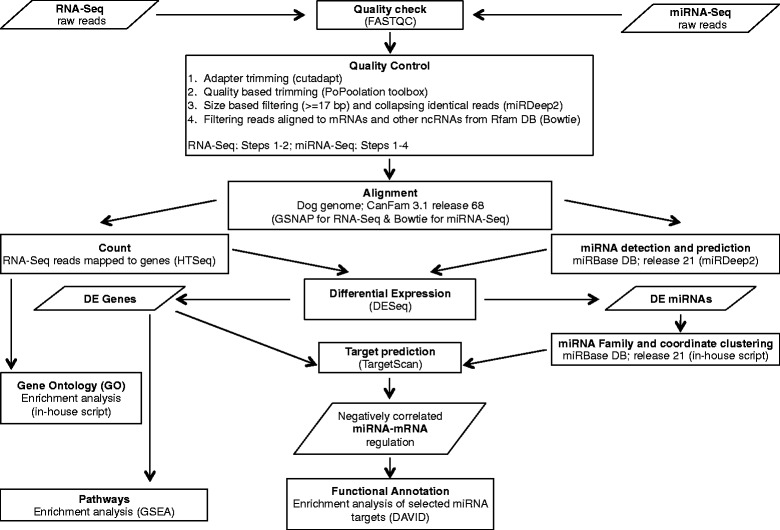
Flowchart of the bioinformatics pipeline. Parallelogram boxes represent input/output. Rectangular boxes represent processing steps. Tools, software and scripts used are mentioned in parentheses

A summary flowchart of the bioinformatics pipeline is presented in Fig. 2. Reads were trimmed to remove adaptor sequences and bad quality regions, and filtered according to length. On average, 96 % of reads were retained after these quality control steps (Additional file 4: Table S1). Quality-controlled reads were mapped to the dog genome (Ensemble’s CanFam3.1; release 68) using GSNAP [55]. On average, 79.3 % of the raw paired-end reads of RNA-Seq data could be uniquely mapped with a proper alignment of the paired ends (Additional file 4: Table S1). Out of 24580 genes annotated in the dog genome, we could identify 12130 (49 %) in our samples (Additional file 5: Table S2). The transcriptome of MDCK and MDCK-Ras cells is available in Additional file 6: S3.

### Differential expression analysis of genes

Sample-to-sample heat map analysis of RNA-Seq data showed a uniform expression pattern among the biological replicates (Additional file 7: Figure S2). Using DeSeq [58], 4705 genes out of 12130 identified genes, were found to be significantly differentially expressed (p < 0.05) between MDCK and MDCK-Ras cells with 2276 genes upregulated in MDCK and 2429 genes upregulated in MDCK-Ras cells (Additional file 5: Table S2). Furthermore, we also report genes with at least two-fold change, which may represent biologically meaningful differential expression levels. The numbers of genes are present in parentheses in Additional file 5: Table S2.

### Validation of RNA-Seq data

We validated selected differentially expressed genes using real time quantitative PCR (RT-qPCR). Table 1 shows expression values obtained by RNA-Seq for genes, which we subsequently validated. For comparison, we included genes known or suspected to be involved in EMT, but also others not yet discussed in the process of Ras induced EMT of MDCK cells. For some genes, mRNA levels in either MDCK or MDCK-Ras were below the detection limit in at least one biological sample (i.e., with a Ct > 35). For these cases, we used a conservative test of differential expression (see Methods).

**Table 1:** RNA-Seq data of genes selected for validation by RT-qPCR. Listed are genes sorted according to their log2 fold change

Gene ID	Gene name	log2 fold change	*p* value
**MDCK:**			
ENSCAFG00000031946	*CLDN2*	−6.259056032	5.47E-12
ENSCAFG00000016608	*SLIT2*	−6.215152318	4.30E-13
ENSCAFG00000013287	*MAPK4*	−6.192289989	1.03E-29
ENSCAFG00000001015	*ANXA13*	−5.819849287	2.37E-08
ENSCAFG00000001531	*SMO*	−5.748090387	1.31E-20
ENSCAFG00000011532	*EGF*	−5.740353485	6.84E-27
ENSCAFG00000001403	*PODXL*	−4.106838157	4.13E-11
ENSCAFG00000010602	*ELF3*	−3.946262741	8.64E-31
ENSCAFG00000020397	*CDH16*	−2.380998977	1.40E-09
ENSCAFG00000020305	*CDH1*	−1.352645238	0.000860681
**MDCK-Ras:**			
ENSCAFG00000007923	*MSC*	7.653897777	9.15E-05
ENSCAFG00000006138	*LUM*	7.532307731	3.38E-10
ENSCAFG00000015054	*MMP1*	7.288819658	2.01 E-07
ENSCAFG00000006766	*DLC1*	7.217480395	6.66E-14
ENSCAFG00000029321	*WNT5A*	7.06017597	6.47E-06
ENSCAFG00000007673	*MMP14*	7.008651621	2.60E-14
ENSCAFG00000002525	*COL15A1*	6.927033269	2.69E-09
ENSCAFG00000011197	*SULF1*	6.905784034	6.41 E-09
ENSCAFG00000005497	*ZEB2*	6.753561154	5.82E-13
ENSCAFG00000017943	*ANXA6*	6.741711305	6.71 E-64
ENSCAFG00000004023	*ZEB1*	6.167816343	7.14E-16
ENSCAFG00000002528	*TGFBR1*	4.97741794	4.72E-37
ENSCAFG00000006638	*SNAI2*	4.785172701	4.54E-07
ENSCAFG00000011499	*SNAI1*	1.367598098	0.035198575

**Fig. 3:**
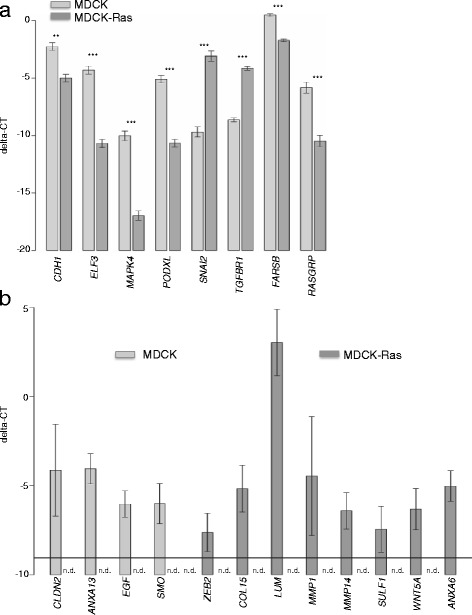
RT-qPCR validation of selected, differentially expressed genes. **a** Differential expression of selected genes validated by RT-qPCR, where all samples were above the detection limit. Shown are delta-Ct values relative to the housekeeping gene, where bars indicate the mean ± standard error margins of four biological replicates; stars indicate significance values: ***: p < 0.001; **: p < 0.01. **b** RT-qPCR of selected genes, where at least one sample was below the detection limit. Depicted are delta-Ct values relative to the housekeeping gene, where bars indicate the mean ± 95 % confidence interval of four biological replicates. n.d.: not detectable. Note that the confidence limits do not overlap the detection limit, i.e., the horizontal line at −9.05

We confirmed enhanced expression of E-Cadherin (*CDH1*), E47-like factor 3 (*ELF3*), Mitogen Activated Protein Kinase 4 (*MAPK4*) and Podocalyxin (*PODXL*). The expression of these genes was high in MDCK cells and strongly reduced in mesenchymal MDCK-Ras cells (Fig. 3a). Furthermore, we validated the increased expression of Claudin2 (*CLDN2*), kidney epithelium specific Annexin13 (*ANXA13*), Epidermal Growth Factor (*EGF*) and Smoothened (*SMO*) in epithelial MDCK cells. These genes were strongly expressed in MDCK cells and not detectable in MDCK-Ras cells (Fig. 3b). Expression of epithelial Cadherin 16 (*CDH16*) and Slit homolog 2 (*SLIT2*) was validated in MDCK cells by RT-qPCR but not detectable in MDCK-Ras cells. The conservative statistical test was not significant (data not shown).

Expression profiling of MDCK-Ras cells clearly showed a mesenchymal gene expression signature. We confirmed enhanced expression of the EMT-TFs *SNAI2* (Fig. 3a) and *ZEB2* (Fig. 3b), of components of the extracellular matrix (ECM), e.g., Collagen 15 (*COL15*), Lumican (*LUM*) and ECM remodeling factors including members of the Matrix Metalloprotease (MMP) family (*MMP1*, *MMP14*) and Sulfatase 1 (*SULF1*) in MDCK-Ras cells compared to MDCK cells (Fig. 3b). Finally, we validated enhanced expression of the TGFβ-Receptor 1 (*TGFBR1*; Fig. 3a), *WNT5A* and Annexin 6 (*ANXA6*) (Fig. 3b) in MDCK-Ras cells compared to MDCK cells. For two low expressed genes, Musculin (*MCS*) and Deleted in liver cancer 1 (*DLC1*), we could validate the increased expression in MDCK-Ras with RT-qPCR, but the conservative test was not significant (data not shown). Importantly, we may conclude from the successful validation with RT-qPCR of a subset of genes that the expression of the remaining genes found in our screen is reliable.

### Gene set enrichment in MDCK and MDCK-Ras cells

After validation of our RNA-Seq data we went further and analysed gene signatures to refine our understanding of Ras induced EMT in MDCK cells.

**Fig. 4:**
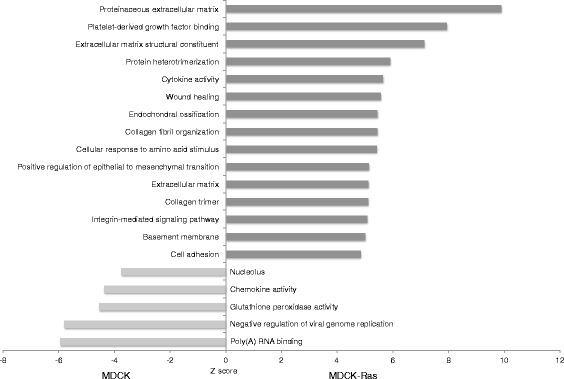
Selected GO terms enriched in MDCK and MDCK-Ras cells. Shown are the GO terms with a false discovery rate (FDR) ≤ 5 %. The z-score represents the normalized average up- or downregulation of genes within the specific GO terms. A positive z-score indicates higher expression in MDCK-Ras cells, a negative z-score a higher expression in MDCK cells

Among GO categories upregulated in MDCK cells, the top five GO terms (false discovery rate (FDR) ≤ 5 %) are “Poly(A) RNA binding”, “Negative regulation of viral genome replication”, “Glutathion peroxidase activity”, “Chemokine activity” and “Nucleolus” (Fig. 4). Conversely, among GO categories upregulated in MDCK-Ras cells, the top 15 GO terms (FDR ≤ 5 %) mainly refer to components of the ECM, e.g., “Proteinaceous extracellular matrix” and “Extracellular matrix structural constituent” (Fig. 4). Furthermore, GO-terms referring to processes involving EMT were significantly enriched, e.g., “Wound healing”, “Integrin mediated signaling pathway” and “Cell adhesion” (Fig. 4)*.*

**Fig. 5:**
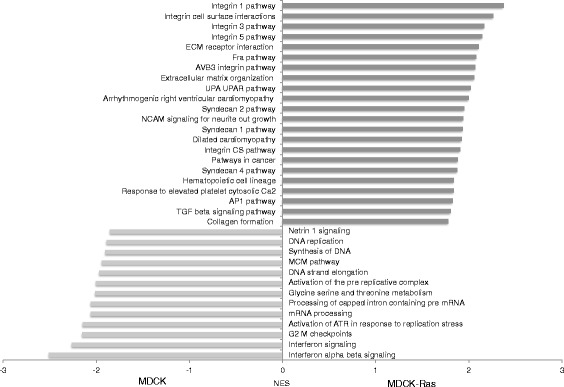
Selected pathways enriched in MDCK and MDCK-Ras cells. Shown are pathways with a false discovery rate (FDR) ≤ 10 %. NES is the Normalized Enrichment Score for up- and downregulated genes belonging to the specific pathways. A positive NES indicates higher expression in MDCK-Ras cells, a negative NES a higher expression in MDCK cells

**Fig. 6:**
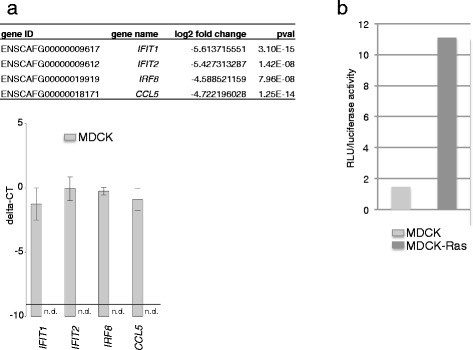
Activation of selected pathways. **a** RNA-Seq data (upper panel) and validation of selected IFN-regulated genes by RT-qPCR (lower panel). **b** Smad2 dependent reporter gene assay shows strong activation of the reporter gene in MDCK-Ras cells compared to MDCK cells

Pathway analysis revealed the upregulation of canonical pathways (REACTOME, KEGG and PID) for steady state metabolism (“G2 M checkpoints”, “mRNA processing”, “Synthesis of DNA”) in MDCK cells (FDR ≤ 10 %; Fig. 5). Interestingly, the most enriched pathways in MDCK cells were “Interferon alpha beta signaling” and “Interferon signaling” (Fig. 5). We selected a group of interferon regulated genes with a log2 fold change >2 and a p-value < 0.05 from our RNA-Seq data (Fig. 6a; upper panel) and validated their expression in MDCK and MDCK-Ras cells. For *IFIT1*, *IFIT2*, *IRF8* and *CCL5* we observed high expression in MDCK cells, while expression was below the detection limit in MDCK-Ras cells (Fig. 6a; lower panel). These differences were significant in spite of the conservative approach when testing.

Pathways upregulated in mesenchymal MDCK-Ras cells (Fig. 5) include mainly those related to integrin signaling (“Integrin 1 pathway”, “Integrin 3 pathway”, “Integrin 5 pathway” and “AVB3 integrin pathway”) and pathways related to ECM (“ECM receptor interaction”, “Extracellular matrix organisation”). We also found enrichment of genes involved in AP1 signaling (“Fra pathway”, “AP1 pathway”) and Plasminogen signaling (“UPA UPAR pathway”). These pathways are known to be crucial for EMT and invasion [49, 68, 69]. Enrichment of the “TGF beta signaling pathway” (Fig. 5) suggests active TGFβ1 signaling in MDCK-Ras cells.

We validated activation of this particular pathway with reporter gene assays. Cells were transfected with a reporter construct containing a Smad response element (SRE) upstream of the luciferase gene. For normalisation we used renilla luciferase. In MDCK cells we detected basal reporter activity (Fig. 6b), probably due to the presence of TGFβ1 in the serum, whereas mesenchymal MDCK-Ras cells show a massive upregulation of the reporter gene (Fig. 6b).

In summary, gene set enrichment analysis revealed GO terms and pathways differentially expressed between epithelial and mesenchymal cells that are implicated in EMT (AP1 pathway, UPA UPAR signaling, TGF beta signaling). Additional pathways particularly reflect the differences in the composition of the ECM and integrin mediated signaling during EMT. Interestingly, we also detected novel GO terms and pathways not yet linked either to MDCK cells (interferon signaling related pathways, “Netrin 1 signaling”) or the process of EMT (“Hematopoietic cell lineage”, “Response to elevated platelet cytosolic calcium”; Fig. 5). The role of these pathways and the genes therein will be the focus of further research. We are confident that, with this information on gene expression, new players and mechanisms in the complex field of EMT will be identified.

### miRNA expression patterns in MDCK and MDCK-Ras cells

For miRNA-Seq, we sequenced four biological replicates of MDCK and MDCK-Ras cells (i.e., in total 8 samples) on an Illumina GAII. Sequencing runs yielded 14–43 million 36 bp single-end reads per sample, with a total of 207 million single-end reads (Additional file 8: Table S3).

### Read quality control and mapping

A summary flowchart of the bioinformatics pipeline is presented in Fig. 2. Adaptor sequences were removed, read quality was checked, and reads were trimmed and filtered for length. This reduced the average read length of our data from 36 bp (originally sequenced) to 22–23 bp, the expected length of mature miRNAs (Additional file 9: Figure S3A). Reads that mapped to other non-coding RNAs (e.g., tRNA, snRNA, snoRNA, scRNA, rRNA, etc.) according to the Rfam database [63] were discarded. On average, about 52 % of the reads were retained after all quality control and filtering steps (Additional file 8: Table S3). Quality controlled reads were aligned to the dog genome (Ensemble’s CanFam3.1; release 68) using Bowtie [64]. Only 2 % of these quality controlled reads got discarded during alignment, such that about 50 % of the raw miRNA-Seq reads could be mapped (Additional file 8: Table S3).

### miRNA detection and prediction

Using miRDeep2 [62] and miRBase (release 21; www.mirbase.org), we found in total 380 miRNAs present in MDCK and/or MDCK-Ras cells of which 219 were known dog miRNAs. Among the remaining 161 miRNAs, 94 were predicted to be homologous to humans and 42 to miRNAs of other species present in miRBase. Twenty five miRNAs were completely novel and not yet described in miRBase (Additional file 10: Table S4). These completely novel miRNAs varied widely in read counts of their predicted mature sequences. Information on mature and precursor sequences, their respective read counts and genome coordinates of these novel miRNAs is presented in Additional file 11: S4. Sequences derived from the other arm of the precurser miRNA (“star sequences”) are degraded quickly, such that their mapped read counts were generally lower than those for predicted mature sequences (Additional file 11: S4). All novel miRNAs make a stable hair-pin structure as predicted by miRDeep2, using the RNA secondary structure prediction algorithm (RNAfold).

For validation with qPCR, we chose the miRNAs with the highest read counts, i.e., novel miRNA#1-3. All three novel miRNAs are located in the introns of different genes in dog (intron 5 of GSN, intron 11 of c5orf165, and intron 17 of RGS3, respectively). In the human orthologs of these genes, no miRNAs are annotated in any of these genes. This further shows that the novel miRNAs are exclusively expressed in dog.

**Fig. 7:**
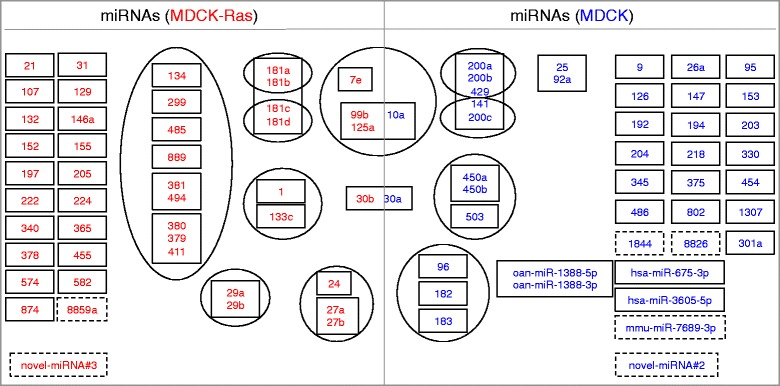
RT-qPCR validation of selected, differentially expressed miRNAs. Depicted are delta-Ct values relative to the internal control (RNU6B), bars indicate means ± standard error margins of four biological replicates (***p < 0.001; **p < 0.01; *p < 0.05)

We performed RT-qPCR analyses for these miRNAs in MDCK and MDCK-Ras cells and included, as negative controls, mouse (EpH4 and RasXT cells; [8, 9]) and human samples (prostate cancer cell lines PC-3 (ATCC: CRL-1435), DU-145 (ATCC:HTB-81) and Ewing’s sarcoma cell lines (A-673 (ATCC-CRL-1598) and TC-71) too. As expected, novel miRNA #1 and novel miRNA # 2 could not be amplified in human and mouse samples (data not shown), but could be amplified in MDCK and MDCK-Ras cells (Fig. 7). With novel miRNA#3, the assay was unspecific. We extended our search for orthologs with bioinformatics analysis.

With the novel miRNAs, we could increase the number of dog miRNAs in miRBase (release 21) by 1.4 times from 453 to 614. Mature and hairpin sequences of newly found miRNAs in the dog will be deposited to miRBase and are expected to be incorporated into the next release of miRBase. The miRNAome of MDCK and MDCK-Ras cells (mapped read counts and miRNAs computed for differential expression) is available in Additional file 12: S5.

### Differential expression analysis of miRNAs

**Fig. 8:**
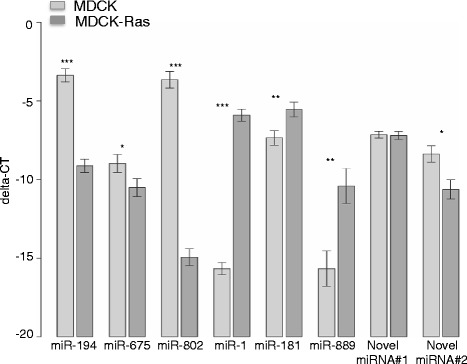
miRNA gene clusters in MDCK and MDCK-Ras cells. Differentially expressed miRNAs grouped with respect to family information (rectangular boxes) from miRBase (release 21) and genome coordinate (ellipses); inter-miRNA distance <10 kb. miRNAs with no family information are presented in rectangular boxes with dashed lines. miRNAs in blue are significantly upregulated in MDCK cells whereas miRNAs in red are significantly upregulated in MDCK-Ras cells. Full names of miRNAs are mentioned for all species except dog

A heat map analysis of miRNA-Seq data comparing the eight samples showed uniform expression pattern within the biological replicates and differences between MDCK and MDCK-Ras cells (Additional file 9: Figure S3B). Using DeSeq [58], 87 miRNAs were found to be differentially regulated (p < 0.05) (Additional file 10: Table S4). Eighty miRNAs were already annotated for dog, of which 36 were expressed in MDCK and 44 in MDCK-Ras. Numbers of miRNAs with a fold change of at least 2 are presented in parentheses in Additional file 10: Table S4. Two of the differentially expressed miRNAs were predicted to be homologous to human miRNAs and three to other species (mouse and platypus). All five miRNAs from human, mouse and platypus were MDCK-specific (Additional file 10: Table S4). Two out of 25 completely novel miRNAs were differentially expressed: one was MDCK-specific, the other significantly higher expressed in MDCK-Ras cells (Additional file 10: Table S4). Differentially expressed miRNAs were grouped into their families based on sequence similarity and according to their proximity in the genome (inter-miRNA distance <10 kb) (Fig. 8).

### miRNA expression in epithelial MDCK cells and mesenchymal MDCK-Ras cells

It has been shown that miRNAs are differentially expressed in the process of EMT in MDCK cells induced by EMT transcription factors [33]. This study utilized MDCK cells individually overexpressing the transcription factors *TWIST1*, *TWIST2*, *SNAI1*, *SNAI2*, *ZEB1*, *ZEB2* and *E47* and the EMT inducer *LOXL2*. Furthermore, the expression pattern of miRNAs and the changes thereof in the process of EMT were analysed [33]. Since this study partially overlaps with our work, we compared our miRNA data obtained with NGS technology with this published dataset obtained with microarray technology (Table 2). Approximately one third of the miRNAs present in MDCK cells in our dataset were seen previously (Table 2; [33]). miRNAs detected in MDCK cells in both analyses included members of the epithelial-specific miR-200 family (miR-200a,b,c, miR-141), and the miR-96, miR-182 and miR-183, which are within a single genomic cluster (Table 2; Fig. 8). In our NGS dataset of miRNAs expressed in MDCK cells we further detected miR-450a/b and miR-503, which belong to a single cluster, and the kidney-specific miR-192 and miR-194 (Table 2; Fig. 8). Additional miRNAs detected exclusively by our NGS analysis or found in both analyses are listed in Table 2. Similarly, we found a clear overlap with miRNAs strongly expressed in MDCK-Ras cells and the data on miRNA expression from MDCK cells overexpressing EMT inducers described in [33] (Table 2, left column). Furthermore, with NGS we found additional miRNAs present in mesenchymal MDCK-Ras cells not previously described in [33] (Table 2, right column). This includes all members of the miR-181 family and the miR-1/133 cluster. Notably, this cluster contains the miRNAs with the highest fold change during EMT (miR-1: log2 fold change ≈ 10; miR-133c: log2 fold change ≈ 8). We next performed RT-qPCR validation of the expression level of selected miRNAs detected with NGS and present exclusively in our dataset (Table 3). For epithelial MDCK cells, we chose miR-194, miR-675, miR-802 and, for mesenchymal MDCK-Ras cells, miR-1, miR-181b and miR-889 (Table 3). Importantly, we could validate the miRNA-Seq pattern of all selected miRNAs (Fig. 7).

**Table 2:** miRNAs differentially expressed during EMT in MDCK cells: Comparison of miRNAs detected in two independent screens. Left column: Common miRNAs detected by microarray in [33] and by NGS in our study; right column: miRNAs detected exclusively by NGS

Common miRNAs detected on miRNA array and by NGS:		miRNAs detected exclusively by NGS:	
MDCK	MDCK-Ras	MDCK	MDCK-Ras
miR-9	miR-129	miR-10a	let-7e
miR-25	miR-132	miR-26a	miR-1
miR-92a	miR-152	miR-30a	miR-21
miR-95	miR-155	miR-126	miR-24
miR-96	miR-224	miR-147	miR-27a
miR-141	miR-340	miR-153	miR-27b
miR-182	miR-411	miR-192	miR-29a
miR-183	miR-455	miR-194	miR-29b
miR-200a	miR-574	miR-301a	miR-30b
miR-200b		miR-345	miR-99b
miR-200c		miR-429	miR-107
miR-203		miR-450a	miR-125a
miR-330		miR-450b	miR-133c
miR-375		miR-454	miR-134
miR-486		miR-503	miR-146a
		miR-675-3p	miR-181a
		miR-802	miR-181b
		miR-1307	miR-181c
		miR-1388-3p	miR-181d
		miR-1388-5p	miR-197
		miR-1844	miR-222
		miR-3605-5p	miR-299
		miR-7689-3p	miR-365
		miR-8826	miR-378
			miR-379
			miR-380
			miR-381
			miR-485
			miR-494
			miR-582
			miR-874
			miR-889
			miR-8859a

**Table 3:** miRNAs differentially expressed during EMT in MDCK cells: miRNAs selected for validation. Expression levels of miRNAs from miRNA-Seq data that were selected for validation with RT-qPCR. miRNAs are sorted according to their log2 fold change

miRNA ID	log2 fold change	*p* value
**MDCK:**		
cfa-miR-194	−4.142446835	1.29E-18
hsa-miR-675-3p	−4.685451438	7.94E-08
cfa-miR-802	−7.829687531	3.70E-26
**MDCK-Ras:**		
cfa-miR-1	10.03297657	7.04E-54
cfa-miR-181b	2.281692113	3.17E-07
cfa-miR-889	6.167513751	4.26E-14

Additionally, we included the novel miRNAs into differential expression analysis of the miRNAs detected in our screen. Two out of these 25 novel miRNAs (novel miRNA#2 and novel miRNA#3) were significantly differentially expressed between MDCK and MDCK-Ras cells. These miRNAs had relatively high mature read counts (Additional file 11: S4). Novel miRNA#2 was higher expressed in MDCK cells than in MDCK-Ras cells; novel miRNA#3 in the reverse fashion. For validation of the novel miRNAs (including the differentially expressed novel miRNAs#2 and #3) we selected the top three miRNAs with the highest read counts. Novel miRNA#1 is equally present in MDCK and MDCK-Ras cells (Fig. 7). This is in accordance with differential expression analysis (Additional file 12: S5). Novel miRNA#2 is significantly higher expressed in MDCK cells compared to MDCK-Ras cells (Additional file 12: S5 and Fig. 7). Differential expression analysis revealed higher expression of novel miRNA#3 in MDCK Ras cells compared to MDCK cells (Additional file 12: S5). Unfortunately, the expression pattern of this miRNA could not been validated, because the RT-qPCR assay was not specific.

### miRNA target prediction

miRNA-target pairs computed by TargetScan [65] were further refined by the expectation that the expression of miRNAs and their target genes is negatively correlated [66]. This resulted in a total of 1975 differentially expressed gene targets. Targets of miRNA-133c were of special interest, as this miRNA was highly and significantly upregulated in MDCK-Ras cells (log2 foldchange = 8.2) and its role in EMT has not yet been studied well. Table 4 summarizes predicted targets of four selected miRNAs. For each miRNA, we show the top 5 targets (bold) and targets of special interest (Table 4). The reduced expression of specific targets of miR-133c (e.g., *ANXA13, EGF, PODXL, CLDN2, ELF3*) in MDCK-Ras cells has already been validated (Fig. 3). Interestingly, miR-133c target genes are annotated for the interferon signaling pathway (e.g., *IFIT1* and IFIT2) (Table 4). Their reduced expression in MDCK-Ras cells was validated by RT-qPCR (Fig. 6). Pathway analysis of its target genes via DAVID [60] showed enrichment (FDR < 5 %) of genes in the Jak-Stat signaling pathway, including several receptors of interleukins (*PTPN6*, *IL22RA1*, *CREBBP*, *IL28RA*, *IL15RA*, *PIK3R5*, *STAT3*; Table 4) and pancreatic cancer pathways (*VEGFC*, *ACVR1B*, *PIK3R5*, *EGF*, *STAT3*; Table 4). Furthermore, targets of miR-1 were computed (Table 4) but these were too few to perform gene set enrichment analysis.

**Table 4:** Predicted gene targets for miR-133, miR-1, novel miRNA#2 and miRNA#3: Top five targets are shown in bold; other targets listed are of special interest

Targets of miR-133c:		Targets of miR-1:	
Gene ID	Gene name	Gene ID	Gene name
**ENSCAFG00000009617**	***IFIT1***	**ENSCAFG00000015835**	***HNRNPU***
**ENSCAFG00000014621**	***MECOM***	**ENSCAFG00000032594**	***PEX12***
**ENSCAFG00000008937**	***KANSL2***	**ENSCAFG00000004084**	***C6orf70***
**ENSCAFG00000017030**	***RANGRF***	**ENSCAFG00000016719**	***SFXN1***
**ENSCAFG00000003692**	***STOM***	**ENSCAFG00000031727**	***STC2***
ENSCAFG00000001015	*ANXA13*	ENSCAFG00000014621	*MECOM*
ENSCAFG00000007357	*ACVR1B*	ENSCAFG00000009612	*IFIT2*
ENSCAFG00000008409	*VEGFC*		
ENSCAFG00000011532	*EGF*		
ENSCAFG00000001403	*PODXL*		
ENSCAFG00000031946	*CLDN2*		
ENSCAFG00000030698	*IL22RA1*		
ENSCAFG00000028524	*IL28RA*		
ENSCAFG00000017387	*PIK3R5*		
ENSCAFG00000010602	*ELF3*		
ENSCAFG00000005213	*IL15RA*		
ENSCAFG00000015213	*STAT3*		
ENSCAFG00000014463	*PTPN6*		
ENSCAFG00000019251	*CREBBP*		
ENSCAFG00000009612	*IFIT2*		
Targets of novel miRNA#2:		Targets of novel miRNA#3:	
Gene ID	Gene name	Gene ID	Gene name
**ENSCAFG00000032470**	***HS2ST1***	**ENSCAFG00000016094**	***FARSB***
**ENSCAFG00000024890**	***NMT2***	**ENSCAFG00000024214**	***USP4***
**ENSCAFG00000001058**	***TXNDC15***	**ENSCAFG00000017895**	***ZNF396***
**ENSCAFG00000012913**	***HDLBP***	**ENSCAFG00000018982**	***MY05B***
**ENSCAFG00000015628**	***SGMS1***	**ENSCAFG00000008674**	***RASGRP1***
ENSCAFG00000001938	*VEGFA*	ENSCAFG00000014009	*CLDN16*
ENSCAFG00000001356	*PDGFB*	ENSCAFG00000010407	*TJP1*
		ENSCAFG00000031946	*CLDN2*

We next performed target prediction for novel miRNA#2. The top 5 targets are shown in bold (Table 4). miRNA#2 targets Vascular Endothelial Growth Factor A (*VEGFA*) and Platelet-derived Growth Factor beta (*PDGFB*) (Table 4). Each of these growth factors promotes EMT [3, 70–72]. In our RNA-Seq data these predicted targets of novel miRNA#2 are significantly downregulated in MDCK cells.

Target prediction of novel miRNA#3 with significantly downregulated genes in MDCK-Ras cells followed by gene set enrichment analysis suggests an influence of this particular miRNA on cell division and growth factor activity. Furthermore, we identified genes of cellular adhesion complexes (e.g., Tight junction protein 1 (*TJP1*) and Claudin 2 (*CLDN2*)) (Table 4), which are targeted by novel miRNA#3. The expression of these targets is low in MDCK-Ras cells as shown by qRT-PCR analysis (Fig. 1 and Fig. 3b). The top five targets are shown in bold (Table 4). FARSB and RASGRP1 were significantly downregulated in MDCK-Ras cells as shown by RT-qPCR (Fig. 3a). Other predicted targets of novel miRNA#3 are significantly downregulated in MDCK-Ras cells in our RNA-Seq data.

## Discussion

In this article, we employed the well known MDCK cell system during Ras induced epithelial-mesenchymal transition (EMT) to complement and extend earlier insights into the transcriptome and miRNAome. RNA-Seq and bioinformatical analysis showed that approximately half of the genes annotated in the dog genome are expressed in MDCK and/or MDCK-Ras cells. With miRNA-Seq, we detected in total 380 miRNAs. 219 are described as dog miRNAs, 161 were newly predicted miRNAs, of which 136 were known in either human or other species and 25 were completely novel.

Among the genes identified with RNA-Seq, approximately one third were differentially expressed between MDCK and MDCK-Ras cells. As expected, expression patterns of genes in MDCK cells show an epithelial-specific signature and those in MDCK-Ras cells a mesenchymal-specific signature**.**

We found that overexpression of oncogenic Ras in MDCK cells induced the combined expression of EMT-TFs. We find that transcription factors of the ZEB (*ZEB1*; *ZEB2*) and snail (*SNAI1*; *SNAI2*) families are highly expressed in MDCK-Ras cells. Because *TWIST1* is not annotated in the CanFam3.1 (release 68), its expression could not be analysed. *TWIST2* had no reads in MDCK cells and too few in MDCK-Ras cells to perform differential expression analyses.

Furthermore, with RNA-Seq we detected a significantly higher expression of *SLIT2* and *SLIT3* in MDCK cells compared to MDCK-Ras cells. In vertebrates, three different slit genes are known, *SLIT1*, *SLIT2* and *SLIT3.* Slit proteins are secreted glycoproteins that bind to receptors of the roundabout (Robo) family [73]. The Slit/Robo signaling pathway is important in axon guidance [73] and has been shown to inhibit signaling by Hepatocyte growth factor (HGF), WNT and Stromal cell-derived factor-1 (SDF-1; also known as CXCL12) [74–76]. Slit2 also acts as a tumor suppressor by maintaining E-Cadherin/β-Catenin functions in breast cancer [75]. Additionally, Slit2 blocks cell motility and tumorigenesis by downregulation of CXCR4 in a mammary tumor model [76]. *SLIT2*, *SLIT3*, and other genes in the gene set “Reactome Netrin 1 signaling” (*DCC*, *UNC5*, *NTN1*, and *NEO1*) are higher expressed in MDCK cells, whereas *CXCR4* is higher expressed in MDCK-Ras cells. Slit and Netrin signaling could therefore contribute to suppress an invasive phenotype in MDCK cells.

Interestingly, genes in pathways related to interferon signaling were upregulated in MDCK cells relative to MDCK-Ras cells and we validated the expression of genes within these pathways (e.g., *IFIT1*, *IFIT2*, *IRF8*, *CCL5*) by RT-qPCR. Ras/MEK signaling has been shown to suppress IFN regulated genes in human cancer cells [77] probably by suppressing IRF1 [78]. Similar to this, expression of *IRF1* and interferon regulated genes in our dataset was significantly higher in MDCK cells compared to MDCK-Ras cells. In MDCK cells we also detected expression of epithelial specific *IL28RA*, which is no longer present after Ras-transformation. Downstream signaling of this receptor for type III interferon [79] is very similar to type I interferon signaling [80] and results in the induction of a similar gene set. The enrichment of interferon regulated genes in MDCK cells might therefore also be due to IL28RA activation. IL28RA acts in a functional complex with IL10R2 [81]. *IL10RB* is expressed in MDCK cells and might cooperate with IL28RA in activating IFN-type III signaling. Furthermore, we found that miR-133c targets many genes within IFN signaling. miR-133c is highly upregulated in MDCK-Ras cells. The expression of IFN signaling related genes in these cells might be suppressed by miR-133c.

Among genes upregulated in MDCK-Ras cells compared to epithelial MDCK cells, TGFβ pathway gene sets were enriched. Complementing this downstream analysis, a strong induction of a reporter gene containing a Smad response element upstream of the luciferase gene was found exclusively in MDCK-Ras. This suggests that MDCK-Ras cells produce bioactive TGFβ1, which is able to stimulate the receptor in an autocrine manner. The upregulation of TGFBRI in MDCK-Ras cells might potentiate this autocrine loop. Nevertheless, we do not exclude the possibility of a constitutive active TGFβ-Receptor in MDCK-Ras cells.

TGFβ1 is a pleiotropic growth factor acting in a context- and cell type-specific manner. In addition to other functions, TGFβ1 also exerts immunoregulatory functions acting on the expression of immune genes [82]. Thus, the enhanced expression of interferon regulated genes in MDCK cells but not in MDCK-Ras cells, may not only be due to direct Ras effects or targeting of these genes by miR-133c but also due to Ras induced TGFβ1 signaling. TGFβ1 also stimulates the synthesis of many extracellular matrix (ECM) proteins and matrix degrading enzymes. In MDCK-Ras cells, mRNA levels of genes involved in ECM remodeling and known to be induced by TGFβ are upregulated, e.g., MMPs, collagens, *FN1* and tenascin-C (*TNC*). Our transcriptome data correspond to published data on the protein level [15, 41].

ECM components signal by binding to integrins located at the cell membrane. The expression of integrins and their ligands is altered by TGFβ1 [83, 84]. Our data show that Ras-induced EMT changes the mRNA expression patterns of integrins dramatically. These changes parallel those described on the protein level [15, 41]. Corresponding to the gene expression pattern of integrins, gene signature analysis showed the presence of many pathways related to the interaction between integrins and ECM in MDCK-Ras cells. Changes in the expression and composition of integrin heterodimers during EMT also modify the impact of growth factor stimuli, the structure of the cytoskeleton and gene expression patterns [3, 85]. Profound changes of integrin heterodimers are described during Ras/TGFβ induced EMT in breast cancer cells [14] and during tumor progression in vivo [85, 86].

An additional pathway capable of inducing EMT is the WNT/ β-Catenin pathway [7]. We detected strong upregulation of *WNT5A*, *WNT5B* and *WNT7A* in MDCK-Ras cells. Similarly, proteomics profiling of Ras/TGFβ induced EMT in MDCK cells shows upregulation of WNT family members during EMT [15]. Additionally we find upregulated expression of the WNT receptors frizzled 2 (*FZD2*) and frizzled 4 (*FZD4*) in MDCK-Ras cells. WNT5A and WNT5B are ligands for and bound by FZD2 and FZD4, and this binding has been shown to drive EMT and is elevated in metastatic tumors [87]. Furthermore, we find upregulation of WNT target genes (e.g., *TCF4*, *SNAI2*, *MYC* and *JUN*) in MDCK-Ras cells which supports the assumption of active WNT signaling in these cells.

Sustained WNT signaling has been shown to contribute to the pathogenesis of kidney fibrosis [88]. We detect upregulation of pro-fibrotic genes, especially *TGFβ1*, collagens, *FN1*, MMPs, integrins and growth factors like *PDGF* and *EGF*. Activation of Ras oncogenes and downstream pathways is also reported in renal fibrosis [89]. Obviously, MDCK-Ras cells (epithelial MCDK cells that had undergone EMT) resemble fibrotic cells and might be used to study aspects of (kidney) fibrosis.

Recently, miRNAs involved in fibrosis have been identified [18, 27, 28]. We could detect the differential expression of pro-fibrotic miRNAs (miR-21, miR-155, miR-27) in MDCK-Ras cells, whereas anti-fibrotic miRNAs (miR-200a/b, miR-141, miR-194, miR-204 and miR-26a) were significantly reduced in MDCK-Ras cells compared to MDCK cells. We find upregulation of the miR-183-96-182 cluster in MDCK cells, probably regulated by Myc [90], which is strongly expressed in MDCK cells. In MDCK-Ras cells miRNAs (miR-134, miR-299, miR-379, miR-380, miR-381, miR-411, miR-485, miR-494 and miR-889) within a single chromosomal region (chromosome 8: 69253808–69284297) were upregulated, suggesting a common regulator. Additionally, we find a strong induction of the miR-1/miR-133 cluster in MDCK-Ras cells. Gene signature analysis of the computationally predicted target genes of miR-133c showed enrichment of “JAK/STAT signaling” and “pancreatic cancer” pathways. The most prominently induced miRNA was miR-1 (log2 fold change >10). Computationally predicted targets of miR-1 were too few to show enrichment of any gene signature. Moreover, miR-1 is annotated as muscle and heart specific [91–93] and induced by IFN-β [94]. IFN-β is not expressed in MDCK-Ras cells, suggesting a different way of induction. In addition, we find a clear upregulation of all members of the miR-181 family. This family of miRNAs is induced by TGFβ1 and promotes breast cancer metastasis [95]. TGFβ1 signaling is active in MDCK-Ras cells thereby possibly stimulating the expression of the miR-181 family. Finally we found completely novel miRNAs (not yet described in any species as per miRBase release 21) and validated their expression. Two of these miRNAs (novel miRNA#2 and novel miRNA#3) were differentially expressed in canine MDCK and MDCK-Ras cells. No orthologs were detected in human and mouse cell lines by RT-qPCR or bioinformatical approaches. Furthermore, with target prediction we show that mesenchymal MDCK-Ras specific novel miRNA#3 targets components of the cell-cell junctions, which were shown to be downregulated in MDCK-Ras cells. The influence of miRNAs on the expression of components of cell-cell junctions has been studied and is reviewed in [96]. Further functional studies of our novel miRNAs will reveal their role and importance in different biological processes.

## Conclusion

We present here the transcriptome and miRNAome of epithelial MDCK and mesenchymal MDCK-Ras cells. In addition to miRNAs known previously to exist in the dog genome, we identified others that were either known from other species or are completely novel. We could confirm the signature of many pathways known to regulate the epithelial and mesenchymal state as well as EMT, in particular TGFβ1 as a central factor involved at different levels of EMT. Additionally, pathways novel for MDCK cells e.g., interferon signaling and slit and netrin signaling, were identified. Our data set and analysis will be useful for people working with MDCK cells not only with focus on epithelial polarity and EMT, but also on other aspects of research utilizing MDCK cells.

## Additional files

Additional file 1: Figure S1. Characterization of MDCK and MDCK-Ras cells: (A) Species-specific PCR of restriction fragment length polymorphism. The lane containing the MDCK-Ras sample was inserted from another part of the same gel. (B) Immunoprecipitation and Western Blot analysis of V12-Ha-Ras. (PDF 3891 kb) Additional file 2: S1. Primer assays used for validation of RNA-Seq data. (XLSX 28 kb) Additional file 3: S2. Primer assays used for validation of miRNA-Seq data. (XLSX 8 kb) Additional file 4: Table S1. RNA-Seq raw, quality controlled and aligned reads. Rows represent samples, where MDCK 1–4 and MDCK-Ras 1–4 refer to the biological replicates. Numbers are paired-end read counts in million. (PDF 34 kb) Additional file 5: Table S2. RNA-Seq gene counts. Listed are number of genes found and differentially expressed. The numbers of genes with a fold change greater than two are shown within parentheses. (PDF 38 kb) Additional file 6: S3. Results of RNA-Seq differential expression analysis. (XLSX 1551 kb) Additional file 7: Figure S2. Heatmap plot and dendogram based on sample-to-sample Euclidean distances between RNA-Seq samples (dark for small distances). MDCK 1–4 represent the biological replicates of MDCK cells, MDCK-Ras 1–4 those of MDCK-Ras cells. (PDF 222 kb) Additional file 8: Table S3. miRNA-Seq raw, quality controlled and aligned reads: Rows represent samples, where MDCK 1–4 and MDCK-Ras 1–4 refer to the biological replicates. Numbers are single-end read counts in million. (PDF 35 kb) Additional file 9: Figure S3. (A) FASTQC quality check report on the read length distribution of miRNA-Seq data after adaptor and quality-based trimming of reads. (B) Heatmap plot and dendogram based on sample-to-sample Euclidean distances between miRNA-Seq samples (dark for small distances). MDCK 1–4 represent the biological replicates of MDCK cells, MDCK-Ras 1–4 those of MDCK-Ras cells. (PDF 253 kb) Additional file 10: Table S4. miRNA-Seq gene counts: Listed are numbers of miRNAs detected/predicted when our miRNA-Seq data were aligned to miRNAs from dog, human or other species. Numbers of differentially expressed miRNAs between MDCK and MDCK-Ras cells are shown in the right two columns. The numbers of genes with a fold change greater than two are shown within parentheses. (PDF 37 kb) Additional file 11: S4. List of completely novel predicted miRNAs with the information about their respective mature, star and precursor sequences, genome coordinates and read counts. (XLSX 37 kb) Additional file 12: S5. Results of miRNA-Seq mapped read counts and differential expression analysis. Please note that data are contained in multiple worksheets. (XLSX 78 kb)
