# Supplementary figures and images for: High-throughput mRNA and miRNA profiling of epithelial-mesenchymal transition in MDCK cells
Source: BMC Genomics. 2015 Nov 16;16:944. doi: 10.1186/s12864-015-2036-9 (PMC4647640; doi:10.1186/s12864-015-2036-9)

A

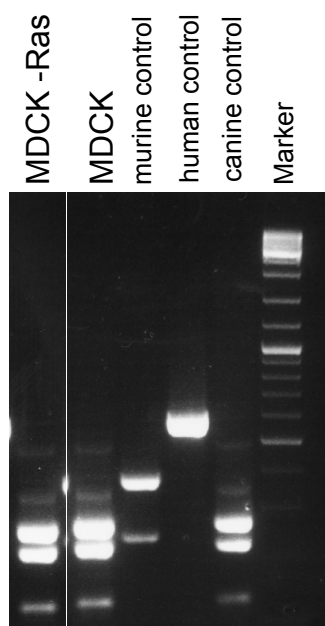

B

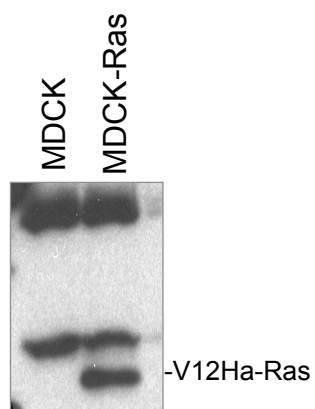

Immunoprecipitation: anti v-Ha-Ras  
Immunoblot: anti panRas V12

Supplement: Additional file 1: Figure S1. — Characterization of MDCK and MDCK-Ras cells: (A) Species-specific PCR of restriction fragment length polymorphism. The lane containing the MDCK-Ras sample was inserted from another part of the same gel. (B) Immunoprecipitation and Western Blot analysis of V12-Ha-Ras. (PDF 3891 kb) [file 12864_2015_2036_MOESM1_ESM.pdf]

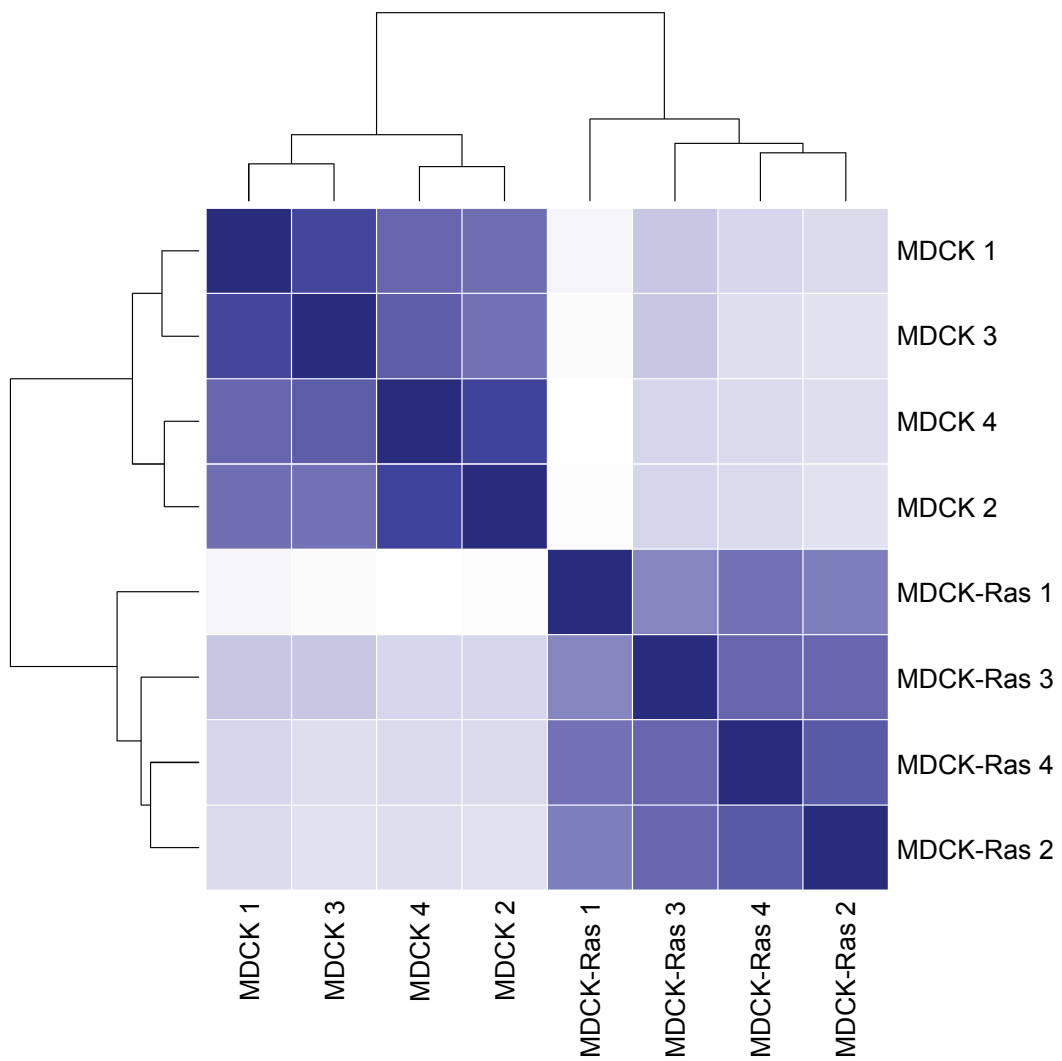

Supplementary Figure S2

Supplement: Additional file 7: Figure S2. — Heatmap plot and dendogram based on sample-to-sample Euclidean distances between RNA-Seq samples (dark for small distances). MDCK 1–4 represent the biological replicates of MDCK cells, MDCK-Ras 1–4 those of MDCK-Ras cells. (PDF 222 kb) [file 12864_2015_2036_MOESM7_ESM.pdf]

A

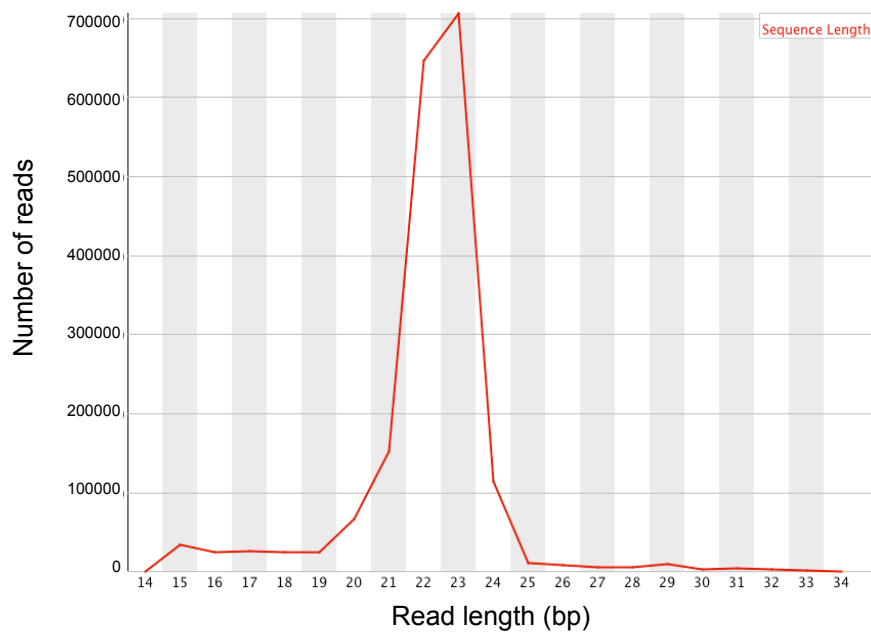

B

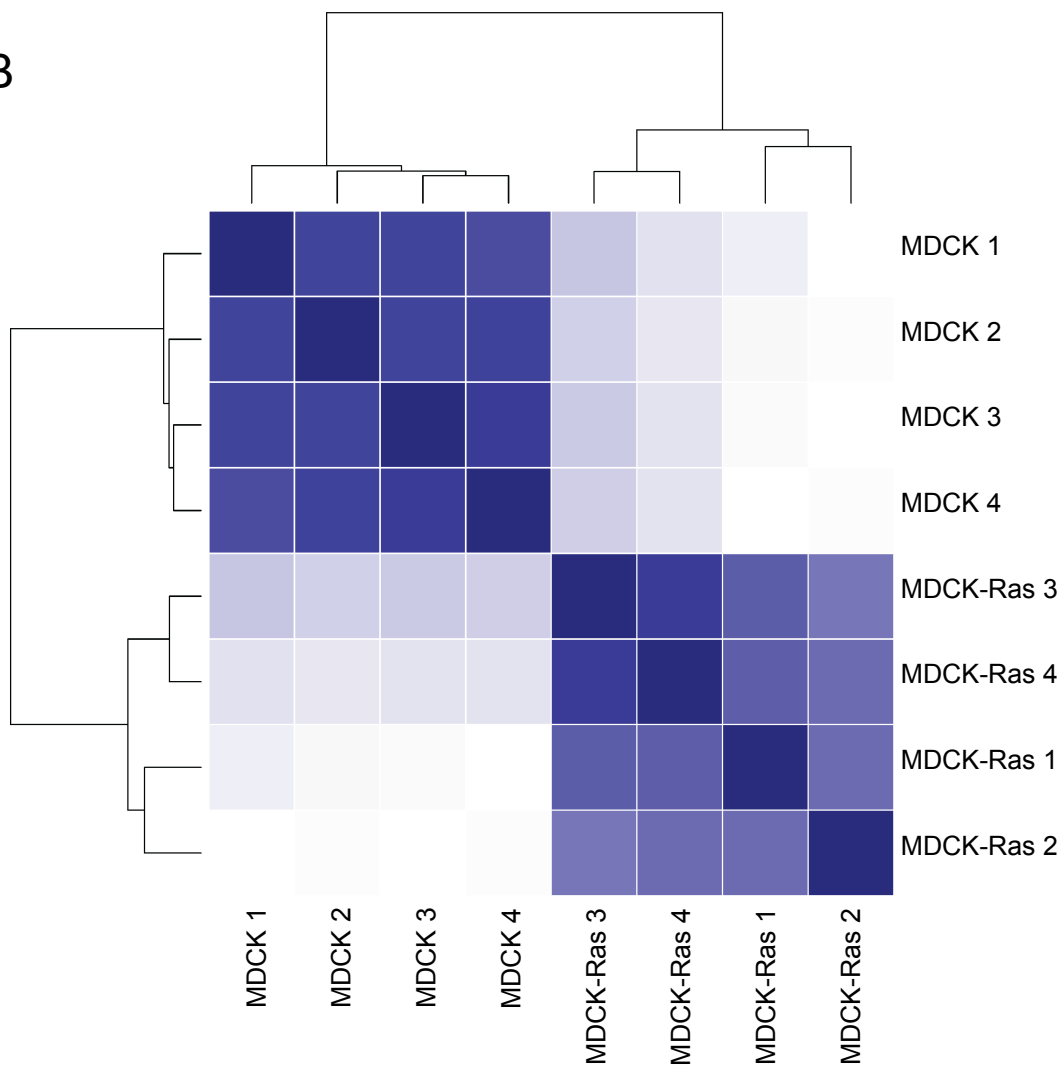

Supplementary Figure S3

Supplement: Additional file 9: Figure S3. — (A) FASTQC quality check report on the read length distribution of miRNA-Seq data after adaptor and quality-based trimming of reads. (B) Heatmap plot and dendogram based on sample-to-sample Euclidean distances between miRNA-Seq samples (dark for small distances). MDCK 1–4 represent the biological replicates of MDCK cells, MDCK-Ras 1–4 those of MDCK-Ras cells. (PDF 253 kb) [file 12864_2015_2036_MOESM9_ESM.pdf]
