# Supplementary material for: High-throughput mRNA and miRNA profiling of epithelial-mesenchymal transition in MDCK cells
Source: BMC Genomics. 2015 Nov 16;16:944. doi: 10.1186/s12864-015-2036-9 (PMC4647640; doi:10.1186/s12864-015-2036-9)
Supplement: Additional file 4: Table S1. — RNA-Seq raw, quality controlled and aligned reads. Rows represent samples, where MDCK 1–4 and MDCK-Ras 1–4 refer to the biological replicates. Numbers are paired-end read counts in million. (PDF 34 kb) [file 12864_2015_2036_MOESM4_ESM.pdf]

Supplementary Table S1: RNA-Seq raw, quality controlled and aligned reads

samples	lane 1	lane 2	lane 3	lane 4	total raw reads	quality controlled reads	reads uniquely mapped in proper pair
MDCK 1	24.2	9.2	22.5	25.4	81.3	78.2	66.7
MDCK 2	8.1	1.6	7.4	8.2	25.3	24.3	20.5
MDCK 3	16.4	6.4	14.9	17.2	54.9	53.0	42.7
MDCK 4	11.8	2.4	10.6	11.8	36.6	35.3	26.3
MDCK-Ras 1	16.4	6.2	14.9	17.2	54.7	52.5	43.4
MDCK-Ras 2	12.5	3.4	11.6	13.0	40.5	38.9	32.5
MDCK-Ras 3	18.3	7.5	16.7	19.3	61.8	59.6	49.4
MDCK-Ras 4	21.1	6.1	19.8	21.5	68.5	65.5	54.5
Total	128.8	42.8	118.4	133.6	423.6	407.3	336.0
