# Supplementary material for: High-throughput mRNA and miRNA profiling of epithelial-mesenchymal transition in MDCK cells
Source: BMC Genomics. 2015 Nov 16;16:944. doi: 10.1186/s12864-015-2036-9 (PMC4647640; doi:10.1186/s12864-015-2036-9)
Supplement: Additional file 5: Table S2. — RNA-Seq gene counts. Listed are number of genes found and differentially expressed. The numbers of genes with a fold change greater than two are shown within parentheses. (PDF 38 kb) [file 12864_2015_2036_MOESM5_ESM.pdf]

Supplementary Table S2: RNA-Seq gene counts.

Total genes in dog genome (CanFam 3 release)	24580
Genes detected in our RNA-Seq data	12130
Significantly differentially expressed genes ($p<0.05$)	4705 (3379)
Significantly differentially expressed MDCK specific genes	2276 (1555)
Significantly differentially expressed MDCK-Ras specific genes	2429 (1824)
