# Supplementary material for: High-throughput mRNA and miRNA profiling of epithelial-mesenchymal transition in MDCK cells
Source: BMC Genomics. 2015 Nov 16;16:944. doi: 10.1186/s12864-015-2036-9 (PMC4647640; doi:10.1186/s12864-015-2036-9)
Supplement: Additional file 8: Table S3. — miRNA-Seq raw, quality controlled and aligned reads: Rows represent samples, where MDCK 1–4 and MDCK-Ras 1–4 refer to the biological replicates. Numbers are single-end read counts in million. (PDF 35 kb) [file 12864_2015_2036_MOESM8_ESM.pdf]

Supplementary Table S3: miRNA-Seq raw, quality controlled and aligned reads:

samples	total raw reads	quality control 1: adaptor removed	quality control 2: quality based trimming	quality control 3: length based filtering	quality control 4: Rfam based filtering	mapped
MDCK 1	32.5	20.2	18.8	18.2	17.5	17.3
MDCK 2	42.5	21.9	21.6	20.7	19.5	19.0
MDCK 3	18.0	9.9	8.9	8.5	8.3	8.1
MDCK 4	22.1	14.3	12.9	12.3	12.0	11.3
MDCK-Ras 1	30.0	16.5	14.7	14.3	14.0	13.9
MDCK-Ras 2	14.1	9.0	8.5	8.2	8.0	6.6
MDCK-Ras 3	25.0	16.9	16.2	15.7	15.4	15.2
MDCK-Ras 4	23.0	14.2	13.0	12.6	12.1	11.8
Total	207.2	122.9	114.6	110.5	106.8	103.2
