# Supplementary material for: High-throughput mRNA and miRNA profiling of epithelial-mesenchymal transition in MDCK cells
Source: BMC Genomics. 2015 Nov 16;16:944. doi: 10.1186/s12864-015-2036-9 (PMC4647640; doi:10.1186/s12864-015-2036-9)
Supplement: Additional file 10: Table S4. — miRNA-Seq gene counts: Listed are numbers of miRNAs detected/predicted when our miRNA-Seq data were aligned to miRNAs from dog, human or other species. Numbers of differentially expressed miRNAs between MDCK and MDCK-Ras cells are shown in the right two columns. The numbers of genes with a fold change greater than two are shown within parentheses. (PDF 37 kb) [file 12864_2015_2036_MOESM10_ESM.pdf]

Supplementary Table S4: miRNA-Seq gene counts

organism	miRBase (release 21)	found (detected/ predicted)	differentially expressed ($p<0.05$)	MDCK	MDCK-Ras
canis	453	219	80 (73)	36 (34)	44 (39)
human	2588	94	2 (2)	2 (2)	0 (0)
rest species	32787	42	3 (3)	3 (3)	0 (0)
novel	-	25	2 (2)	1 (1)	1 (1)
